# Dairy intake during adolescence and risk of colorectal adenoma later in life

**DOI:** 10.1038/s41416-020-01203-x

**Published:** 2021-01-04

**Authors:** Katharina Nimptsch, Dong Hoon Lee, Xuehong Zhang, Mingyang Song, Maryam S. Farvid, Leandro F. M. Rezende, Yin Cao, Andrew T. Chan, Charles Fuchs, Jeffrey Meyerhardt, Jonathan A. Nowak, Walter C. Willett, Shuji Ogino, Edward Giovannucci, Tobias Pischon, Kana Wu

**Affiliations:** 1 Molecular Epidemiology Research Group, Max Delbrück Center for Molecular Medicine in the Helmholtz Association (MDC), Berlin, Germany; 2 Department of Nutrition, Harvard T.H. Chan School of Public Health, Boston, MA USA; 3 Channing Division of Network Medicine, Department of Medicine, Brigham and Women’s Hospital, and Harvard Medical School, Boston, MA USA; 4 Department of Epidemiology, Harvard T.H Chan School of Public Health, Boston, MA USA; 5 Division of Gastroenterology, Massachusetts General Hospital and Harvard Medical School, Boston, MA USA; 6 Clinical and Translational Epidemiology Unit, Massachusetts General Hospital and Harvard Medical School, Boston, MA USA; 7 Universidade Federal de São Paulo, Escola Paulista de Medicina, Departamento de Medicina Preventiva, Sao Paulo, SP Brazil; 8 Division of Public Health Sciences, Department of Surgery, Washington University School of Medicine, St Louis, MO USA; 9 Alvin J. Siteman Cancer Center, Washington University School of Medicine, St Louis, MO USA; 10 Broad Institute of MIT and Harvard, Cambridge, MA USA; 11 Department of Immunology and Infectious Diseases, Harvard T.H. Chan School of Public Health, Boston, MA USA; 12 Yale Cancer Center, New Haven, CT USA; 13 Department of Medicine, Yale School of Medicine, New Haven, CT USA; 14 Smilow Cancer Hospital, New Haven, CT USA; 15 Department of Medical Oncology, Dana-Farber Cancer Institute, Boston, MA USA; 16 Program in MPE Molecular Pathological Epidemiology, Department of Pathology, Brigham and Women’s Hospital, and Harvard Medical School, Boston, MA USA

**Keywords:** Colorectal cancer, Cancer epidemiology

## Abstract

**Background:**

Higher dairy intake during adulthood has been associated with lower colorectal cancer risk. As colorectal carcinogenesis spans several decades, we hypothesised that higher dairy intake during adolescence is associated with lower risk of colorectal adenoma, a colorectal cancer precursor.

**Methods:**

In 27,196 females from the Nurses’ Health Study 2, aged 25–42 years at recruitment (1989), who had completed a validated high school diet questionnaire in 1998 and undergone at least one lower bowel endoscopy between 1998 and 2011, logistic regression for clustered data was used to calculate odds ratios (ORs) and 95% confidence intervals (CI).

**Results:**

Colorectal adenomas were diagnosed in 2239 women. Dairy consumption during adolescence was not associated with colorectal adenoma risk (OR highest vs. lowest [≥4 vs. ≤1.42 servings/day] quintile [95% CI] 0.94 [0.80, 1.11]). By anatomical site, higher adolescent dairy intake was associated with lower rectal (0.63 [0.42, 0.95]), but not proximal (1.01 [0.80, 1.28]) or distal (0.97 [0.76, 1.24]) colon adenoma risk. An inverse association was observed with histologically advanced (0.72 [0.51, 1.00]) but not non-advanced (1.07 [0.86, 1.33]) adenoma.

**Conclusions:**

In this large cohort of younger women, higher adolescent dairy intake was associated with lower rectal and advanced adenoma risk later in life.

## Background

As colorectal carcinogenesis spans several decades, early life exposures may also play a role in colorectal cancer (CRC) development.^1,2^ However, the majority of observational studies have focused on exposures during mid- to late adulthood, capturing predominantly late-onset cases. The recent increase in sporadic early-onset CRC incidence in high-income countries further supports an involvement of early-life behavioural exposures in colorectal carcinogenesis.^3^

There is substantial evidence that higher intake of dairy and calcium during adulthood is protective against CRC, particularly left-sided cancers.^4–6^ Data from the National Health Examination Survey (NHANES) showed that between 2003 and 2006 only 42% of adolescent boys and 13% of girls in the US met their Recommended Dietary Allowance (RDA) for calcium, which may reflect a low intake of dairy products.^7–9^ Only two studies have previously investigated the association between dairy intake in early-life and risk of CRC, both of which had methodological limitations, and results were inconsistent.^10,11^ Moreover, to the best of our knowledge, no study has examined these associations for adenomas, an established CRC precursor.^12^ Our objective was to investigate the association between dairy intake during adolescence and risk of colorectal adenoma in a large, prospective cohort of younger women, the Nurses’ Health Study 2 (NHS2), controlling for relevant early-life and adult behavioural factors, such as early-life physical activity and dairy intake during adulthood.

## Methods

### Study population

The NHS2 is a prospective cohort study of 116,671 female registered nurses aged 25–42 years at enrolment in 1989 who responded to a mailed questionnaire on lifestyle and medical history.^13^ Information on lifestyle, medical history, and newly diagnosed diseases are updated biennially. Starting in 1991, food frequency questionnaires (FFQs) were administered every 4 years to ascertain habitual dietary intake. In 1998, 47,355 women returned a 124-item high school food frequency questionnaire (HS-FFQ). At the time of completion of the HS-FFQ, participants were between 34 and 51 years old. There were no substantial differences regarding baseline characteristics, including dietary intake and CRC risk factors comparing women who had indicated willingness to complete the HS-FFQ and those who did not.^14^ This study was approved by the Institutional Review Boards of the Brigham and Women’s Hospital and the Harvard T. H. Chan School of Public Health and of participating registries as required. Further information, including the procedures to obtain and access data from the Nurses’ Health Studies is described at https://www.nurseshealthstudy.org/researchers (contact email: nhsaccess@channing.harvard.edu).

### Ascertainment of outcome

Colorectal adenomas are usually asymptomatic and therefore typically detected during a lower bowel endoscopic procedure. On each biennial follow-up questionnaires, participants reported whether they had undergone a lower bowel endoscopy (sigmoidoscopy or colonoscopy), the reason for endoscopy (screening or symptoms) and whether polyps had been diagnosed during the procedure. Informed consent to retrieve endoscopy and pathology reports was obtained from the study participants. After obtaining consent from the participants, investigators blinded to exposure status confirmed the reported polyp diagnoses based on medical records, and extracted information on size, number, anatomical location and histology. For the present analysis, we included women who had reported their adolescent diet on the HS-FFQ in 1998 and had undergone at least one lower bowel endoscopy during the study period, i.e. between the return of the HS-FFQ and May 31st, 2011. After excluding women who reported a previous cancer diagnosis (other than non-melanoma skin cancer), ulcerative colitis, Crohn’s disease, or adenomas before completion of the HS-FFQ, 27,196 women remained for analysis. Colorectal adenomas were classified based on their location into proximal colon (cecum, ascending colon, hepatic flexure, transverse colon, splenic flexure), distal colon (descending colon, sigmoid colon) and rectum (including recto-sigmoid junction) adenomas. We further subclassified adenoma into advanced adenoma (defined as adenoma with any of the following features; ≥1 cm in size, tubulovillous histology, villous histology, high-grade dysplasia) and non-advanced adenoma. We also analysed associations by the multiplicity of polyps (single vs multiple).

### Assessment of diet

#### High school diet

Adolescent diet, i.e. dietary intake during high school grades 9–12 (age 13–17 years) was inquired on the 124-item HS-FFQ in 1998. The HS-FFQ was specifically designed to include food items that were typically consumed during the time period between 1960 and 1981, i.e. when the NHS2 participants attended high school. Participants were asked to indicate how often they consumed a specified amount of each of the food items on average, with possible frequencies ranging from “never or less than once per month” to “six or more per day”.

The HS-FFQ has shown moderate-to-good reproducibility in recalling adolescent diet among 333 randomly selected NHS2 participants who completed the same HS-FFQs twice after a 4-year interval.^15^ Mean Spearman rank correlations between the first and second administration of the HS-FFQ was good for the group of dairy products (*r* = 0.64), and milk intake was among the foods with the highest reproducibility (*r* = 0.76). Similarly, the correlation for calcium was *r* = 0.73.

Furthermore, the HS-FFQ has shown reasonable validity when comparing FFQs administered to 80 adolescents aged 13–18 years with the HS-FFQ completed by the same 80 participants 10 years later. The average corrected correlation for 25 nutrients was *r* = 0.58 (range 0.40–0.88) and the correlation for calcium was *r* = 0.84.^16^

#### Adult diet

Habitual dietary intake during adulthood was first assessed in 1991 and every 4 years thereafter using validated FFQs with ~131 food items. Results from reproducibility and validity studies of the adult FFQ have been reported previously.^17–19^ The Pearson correlation coefficients between multiple dietary records and the FFQ ranged for dairy products from 0.57 (hard cheese) to 0.94 (yogurt).^19^

#### Dairy intake

The dairy items on the HS-FFQ included milk (whole, low-fat, non-fat), chocolate milk, instant breakfast drink, yogurt, cottage or ricotta cheese, cheese, cream cheese, ice cream, sherbet, milk shake or frappe. Dairy items on the adult FFQs included skim or low-fat milk, whole milk, cream, sour cream, sherbet/ice milk/frozen yogurt, ice cream, yogurt, cottage/ricotta cheese, cream cheese, other cheese. Of note, milk intake during high school was primarily from whole milk (median (25th, 75th percentile) 1.43 (2.57, 0.86) servings per day for whole milk and 0 (0, 0) servings per day for skim milk), while milk intake during adulthood was primarily from skim milk (median (25th, 75th percentile) 0, (0, 0) for whole milk and 0.81 (0.36, 1.50) servings/day for skim milk). Therefore, associations were not studied separately for adolescent whole vs. skim milk intake.

#### Calcium intake

To investigate whether calcium intake may explain observed associations between adolescent dairy intake and risk of colorectal adenoma, we also calculated intake of calcium (total calcium, dairy calcium, non-dairy calcium) from the HS-FFQ and the adult questionnaires. Calcium intake during adulthood is comprised of calcium from dietary sources and calcium supplements, which is fairly common among adult women in the NHS2, particularly among those with lower dairy intake (for example, in 1999, 49% of the participants with adult dairy intake ≤1 serving/day were calcium supplement users, compared with 42% calcium supplement users in participants with dairy intake ≥3 servings/day, *χ*^2^ *P* < 0.0001). The HS-FFQ did not include questions on calcium supplementation. However, between 1960 and 1980 calcium supplementation was uncommon in adolescents, thus it is safe to assume that calcium intake during adolescence is mostly from dietary, particularly dairy sources only.

Both adult and high school nutrient intakes were energy adjusted utilising the residual method.^20^ To best represent long-term intake during adulthood and to minimise random intra-individual variation, we calculated cumulative average intakes of dairy and nutrients using information from all available adult FFQs up to 2 years prior to the most recent endoscopy.^21^

### Statistical analysis

We used a period-based analytical approach, creating risk sets of separate records for each two-year follow-up period during which a participant underwent a lower bowel endoscopy.^22^ Thus, study participants who underwent several endoscopies during follow-up appear in multiple risk sets. Once an adenoma was diagnosed, participants were censored for all later follow-up cycles. We used multivariable-adjusted logistic regressions for clustered data (each participant poses a cluster) to calculate odds ratios (ORs) and 95% confidence intervals (CI). This analysis method accounts for multiple lower bowel endoscopies and reduces bias due to temporal changes in exposures and covariables.^23–25^

We created a basic model that was adjusted for age, time period of endoscopy, number of reported endoscopies, time in years since most recent endoscopy and reason for current endoscopy (screening vs. symptoms). In the fully adjusted model, we additionally included potential confounders during adolescence and adulthood, such as established CRC risk factors^26^ related to body stature, diet and lifestyle: BMI at age 18 years (<18, 18–20.9, 21–22.9, 23–24.9, ≥25 kg/m^2^), current physical activity (<21, 21–<30, 30–<39, 39–<54, ≥54 MET hours/week), physical activity during 9th–12th grades (quintiles),^27^ current alcohol intake (<5, 5–9.9, 10–14.9, 15–29.9, 30+ g/d), pack-years of smoking (never, 1–4.9, 5–19.9, 20–39.9, 40+ pack-years), regular aspirin use (≥2 tablets/week vs. <2/week), menopausal status/postmenopausal hormone use (premenopausal, postmenopausal with current hormone use, postmenopausal without current hormone use), family history of CRC, total calories during high school (quintiles) and intake of unprocessed red meat and processed meat during high school (quintiles).

Additional adjustment for pack-years of smoking before age 20 years, folate intake during high school, or history of diabetes did not change risk estimates substantially, and these variables were not included in the final models (data not shown). To assess whether observed associations for adolescent dairy intake were independent from dairy intake during adulthood, our multivariable models were adjusted for adult dairy intake. We also analysed data separately by source of dairy (milk, cheese and yogurt), and low vs. high-fat dairy.

To assess whether higher calcium intake may contribute to the observed inverse associations,^6,26,28^ we conducted several sensitivity analyses by (1) investigating the association of total, dairy and non-dairy calcium intake during high school with risk of adenoma and (2) adding total calcium, non-dairy and dairy calcium intake separately to the multivariable models for dairy intake.

Test for trend was performed by including the median intake of each exposure category or quantile as a continuous variable into the models and calculating a *p*-value using the Wald test. We also analysed associations stratified by age at diagnosis, family history of CRC, reason of endoscopy (screening vs. symptoms) as well as risk factors for colorectal neoplasia during early life, i.e., body mass index (BMI) at age 18 years, physical activity during 9th–12th grades, and ever vs. never smoking before age 20 years in order to assess whether these factors modify observed associations. Interactions were assessed by including a cross product term of both exposures [stratification variable and median intake in each category (same as trend variable) as continuous variable for dairy intake] into the models and utilising the Wald test to assess statistical significance. A two-sided *p*-value of less than 0.05 was considered statistically significant.

## Results

Baseline characteristics of NHS2 participants at the time when they returned the HS-FFQ are shown in Table 1. BMI in 1998 and at age 18 years did not differ substantially, while adult attained height slightly increased across quintiles of dairy intake during high school. Women with higher dairy intake tend to have lower pack-years of smoking before age 20, were less likely to be current smokers, and more likely to be physically active in 1998 and during adolescence than women with lower dairy intake. During follow-up, 2239 women were diagnosed with at least one colorectal adenoma (mean age at diagnosis was 52.8 ± 4.4 years, range 37–64 years). 1278 adenomas were classified as non-advanced and 516 as advanced. Based on location, 1128 adenomas were diagnosed in the proximal colon, 959 in the distal colon, and 367 in the rectum. Dairy intake during adolescence was higher (median 2.71 servings/day, 25th percentile 1.63, 75th percentile 3.71) than during adulthood (median 1.96 servings/day, 25th percentile 1.32, 75th percentile 2.78), and dietary intake during adolescence and adulthood were only modestly correlated (Spearman partial correlation corrected for age *r* = 0.38). On the other hand, correlation between adolescent dairy and adolescent calcium intake was high (Spearman correlation *r* = 0.77).

**Table 1:** Age-standardised characteristics in 1998 by quintiles of dairy intake during high school, NHS2.

	Dairy intake during high school				
	Q1	Q2	Q3	Q4	Q5
*Quintile range (servings/day)*	≤1.42	1.43–2.20	2.21–3.14	3.15–3.99	≥4.00
*N*	5425	5397	5525	5756	5094
Age (years)	43.4 (4.3)	43.0 (4.2)	43.2 (4.2)	43.4 (4.1)	42.9 (4.1)
Height (inches)	64.6 (2.6)	64.8 (2.6)	64.9 (2.6)	65.1 (2.5)	65.3 (2.6)
BMI (kg/m^2^)	26.0 (5.7)	25.9 (5.9)	25.8 (5.8)	25.6 (5.6)	25.5 (5.7)
BMI at age 18 years (kg/m^2^)	21.2 (3.2)	21.3 (3.3)	21.2 (3.3)	21.1 (3.1)	21.0 (3.0)
Current smokers (%)	8.4	9.5	7.9	7.1	6.5
Packyears of smoking (among smokers)	14.1 (11.4)	14.2 (11.8)	13.5 (11.1)	12.7 (10.4)	12.7 (10.8)
Packyears of smoking before age 20 years (among smokers)	2.8 (2.2)	2.8 (2.1)	2.7 (2.1)	2.8 (2.2)	2.8 (2.1)
Physical activity (MET-hours/week)	18.1 (23.2)	18.7 (22.4)	19.1 (24.2)	18.8 (22.1)	20.2 (22.9)
Physical activity during 9th–12th grade (MET-hours/week)	46.8 (34.8)	49.3 (35.1)	50.9 (35.8)	52.0 (35.6)	56.6 (37.8)
Family history of colorectal cancer (%)	7.7	8.0	8.7	8.0	8.1
Diabetes (%)	4.6	4.1	4.0	3.7	3.3
Premenopausal (%)	86.8	88.4	88.2	89.3	89.9
Regular aspirin use (%)	10.5	10.0	9.9	11.0	10.8
Screening as indication for the current endoscopy (%)	27.5	25.6	25.8	25.6	26.1
Dietary intake during high school					
Total calcium intake during high school (mg/day)	726 (171)	887 (161)	1111 (286)	1289 (240)	1413 (304)
Dairy calcium intake during high school (mg/day)	413 (175)	587 (167)	827 (321)	1010 (262)	1124 (328)
Non-dairy calcium intake during high school (mg/day)	313 (75)	300 (62)	283 (67)	279 (61)	290 (62)
Folate intake during high school (µg/day)	307 (100)	313 (95)	317 (89)	324 (91)	333 (93)
Red (without processed) meat intake during high school (servings/day)	0.9 (0.4)	0.9 (0.4)	0.9 (0.4)	1.0 (0.4)	1.0 (0.4)
Processed meat intake during high school (servings/day)	0.5 (0.4)	0.6 (0.4)	0.6 (0.4)	0.6 (0.4)	0.7 (0.5)
Dietary intake during adulthood					
Total dairy (servings/day)	1.6 (0.9)	1.9 (0.9)	2.1 (1.0)	2.4 (1.1)	2.7 (1.2)
Total calcium (mg/day)	1356 (685)	1372 (641)	1431 (635)	1495 (636)	1525 (615)

All values other than age have been directly standardised to age distribution (5-year age groups) of all participants who had an endoscopy between 1998 and 2011. Mean (SD) presented for continuous variables.

*BMI* body mass index, *MET* metabolic equivalent task.

### Dairy intake during high school and adenoma later in life

Dairy consumption during adolescence was not significantly associated with risk of colorectal adenoma in the basic model (OR highest [≥4 servings/day] vs. lowest [≤1.42 servings/day] quintile 1.02, 95% CI 0.89, 1.18), the fully adjusted model without (OR 0.98, 95% CI 0.84, 1.15), or after further adjustment for dairy consumption during adulthood (OR 0.94, 95% CI 0.80, 1.11, Table 2). By anatomical subsite, dairy consumption during high school was not associated with risk of proximal or distal adenoma, but a statistically significant inverse association was observed for rectal adenoma (OR 0.63, 95% 0.42, 0.95). A borderline significant inverse association was found for advanced adenoma (OR 0.72, 95% CI 0.51, 1.00), whereas no association was observed for non-advanced adenoma.

**Table 2:** Odds Ratios (ORs) and 95% confidence intervals (95% CIs) for risk of colorectal adenoma according to quintiles of dairy intake during high school, NHS2 1998–2011.

	Dairy intake during high school (servings/day)					
	Q1	Q2	Q3	Q4	Q5	P_trend_
	≤1.42	1.43–2.20	2.21–3.14	3.15–3.99	≥4.00	
*All adenomas*						
N cases	446	420	480	476	417	
Age-adjusted*	1 (Reference)	0.96 (0.84, 1.10)	1.07 (0.93, 1.22)	1.01 (0.89, 1.16)	1.02 (0.89, 1.18)	0.52
Multivariable^†^	1 (Reference)	0.92 (0.80, 1.06)	1.02 (0.88, 1.17)	0.96 (0.83, 1.11)	0.98 (0.84, 1.15)	0.94
Multivariable^†^ plus adult diet	1 (Reference)	0.91 (0.79, 1.05)	1.00 (0.86, 1.15)	0.93 (0.80, 1.08)	0.94 (0.80, 1.11)	0.64
*Proximal adenomas*						
N cases	217	215	226	264	206	
Multivariable^†^ plus adult diet	1 (Reference)	0.96 (0.79, 1.18)	0.99 (0.80, 1.21)	1.08 (0.88, 1.33)	1.01 (0.80, 1.28)	0.56
*Distal adenomas*						
N cases	195	185	200	195	184	
Multivariable^†^ plus adult diet	1 (Reference)	0.93 (0.75, 1.15)	0.97 (0.78, 1.20)	0.90 (0.71, 1.13)	0.97 (0.76, 1.24)	0.79
*Rectal adenomas*						
N cases	76	63	103	72	53	
Multivariable^†^ plus adult diet	1 (Reference)	0.80 (0.57, 1.12)	1.23 (0.89, 1.70)	0.79 (0.55, 1.13)	0.63 (0.42, 0.95)	0.04
*Small, tubular adenomas (non-advanced)*						
N cases	239	243	263	285	248	
Multivariable^†^ plus adult diet	1 (Reference)	0.97 (0.80, 1.18)	1.02 (0.84, 1.24)	1.04 (0.86, 1.27)	1.07 (0.86, 1.33)	0.39
*Large or villous adenomas (advanced)*						
N cases	114	97	122	99	84	
Multivariable^†^ plus adult diet	1 (Reference)	0.83 (0.63, 1.10)	1.01 (0.76, 1.33)	0.76 (0.56, 1.04)	0.72 (0.51, 1.00)	0.05
*Single adenoma*						
N cases	353	329	370	369	342	
Multivariable^†^ plus adult diet	1 (Reference)	0.90 (0.76, 1.05)	0.96 (0.81, 1.13)	0.89 (0.75, 1.05)	0.95 (0.79, 1.14)	0.64
*Two or more adenomas*						
N cases	88	87	108	103	68	
Multivariable^†^ plus adult diet	1 (Reference)	0.99 (0.73, 1.34)	1.22 (0.91, 1.65)	1.13 (0.83, 1.55)	0.90 (0.63, 1.29)	0.88

*Adjusted for age, time period of endoscopy, number of reported endoscopies, time in years since most recent endoscopy and reason for current endoscopy.

^†^Additionally adjusted for BMI at age 18 years (<18, 18–20.9, 21–22.9, 23–24.9, ≥25 kg/m^2^), current physical activity (<21, 21–<30, 30–<39, 39–<54, ≥54 MET hours/week), physical activity during 9th–12th grades (quintiles), current alcohol intake (<5, 5–9.9, 10–14.9, 15–29.9, 30+ g/d), pack-years of smoking (never, 1–4.9, 5–19.9, 20–39.9, 40+ pack-years), regular aspirin use (≥2 tablets/week vs. <2/week), menopausal status/postmenopausal hormone use (premenopausal, postmenopausal with current hormone use, postmenopausal without current hormone use), family history of colorectal cancer, total calories during high school (quintiles) and intake of unprocessed red meat and processed meat during high school (quintiles).

plus adult diet = adult calorie (quintiles) and total dairy intake (quintiles).

To examine whether our results are due to an overall healthier vs. unhealthier diet, we included adolescent dietary pattern in our final models, namely the prudent and the western dietary pattern as reported previously.^29^ Inverse associations between dairy intake and rectal and advanced adenoma were similar after adjustment for prudent dietary pattern (highest vs. lowest quintile rectal: OR 0.63, 95% CI 0.42, 0.94; advanced: OR 0.71, 95% CI 0.51,1.00), but were slightly attenuated after adjustment for western pattern (rectal: OR 0.71, 95% CI 0.47, 1.07; advanced: OR 0.75, 95% CI 0.53, 1.05).

### Individual dairy foods and adenoma

When analysed separately by individual dairy foods, higher milk consumption during high school was borderline significantly associated with lower risk of advanced adenoma (OR comparing ≥4–5 servings per day vs. ≤2–4 servings per week 0.73, 95% CI 0.53, 1.00) and rectal adenoma (OR 0.72, 95% CI 0.50, 1.02) (Supplementary Table 1). Higher yogurt intake during high school (≥1 serving/week vs. never or less than 1 serving/month) was borderline significantly associated with lower risk of multiple adenomas (OR 0.61, 95% CI 0.37, 1.00), while inverse, but not statistically significant, associations were observed for total, proximal, distal, rectal, advanced, and non-advanced adenoma. When associations were examined separately for high- vs. low-fat dairy intake, observed inverse associations for rectal adenoma appeared to be stronger for high-fat (OR highest vs. lowest quintile 0.66, 95% CI 0.45, 0.96,) than for low-fat dairy intake (OR 0.97, 95% CI 0.66, 1.41; Supplementary Table 2).

### Role of calcium intake on observed associations

Total calcium intake during high school was not associated with risk of total (OR highest vs. lowest quintile 1.00, 95% CI 0.87, 1.15) or rectal adenoma (OR 0.91, 95% CI 0.66, 1.26), but a non-significant inverse association was observed with advanced adenoma (OR 0.77, 95% CI 0.57, 1.04). Associations between dairy and non-dairy calcium intake during high school and rectal and advanced adenoma were similar to those observed for dairy intake during high school, but did not reach statistical significance (OR highest vs. lowest quintile, dairy calcium: 0.77, 95% CI 0.54, 1.09 for rectal and 0.78, 95% CI 0.57, 1.07 for advanced adenoma; non-dairy calcium: 0.77, 95% CI 0.52, 1.14 for rectal and 0.88, 95% CI 0.64, 1.23 for advanced adenoma).

When the models were additionally adjusted for calcium intake, inverse associations between dairy intake and rectal adenoma persisted after adjusting for non-dairy calcium intake during high school (OR 0.57, 95% CI 0.38, 0.87), but was no longer statistically significant after adjusting for total (OR 0.59, 95% CI 0.31, 1.11) or dairy calcium intake during high school (OR 0.64, 95% CI 0.33, 1.22, Supplementary Table 3). For advanced adenoma, associations were attenuated and no longer statistically significant after additional adjustment for total (OR 0.81, 95% CI 0.46, 1.43) and dairy calcium (OR 0.88, 95% CI 0.49, 1.59) intake during high school, but remained similar and statistically significant after adding non-dairy calcium to the multivariable models (OR 0.67, 95% CI 0.48, 0.95).

### Joint association of dairy intake during adolescence and adulthood

For a joint analysis of dairy intake during adolescence and adulthood, we created four categories with the cut-offs for low vs. high intake corresponding to the currently recommended intake for both adults and adolescents (3 servings/day).^30^ Compared with low dairy intake during both adolescence and adulthood (*n* = 285 cases), the lowest risk for advanced adenoma was observed for women with high adolescent, but low adult dairy intake (OR 0.79, 95% CI 0.62, 1.00, *n* = 120 cases), followed by women with high dairy intake during both adolescence and adulthood (OR 0.87, 95% CI 0.64, 1.18, *n* = 63 cases), but no association was found for women with low adolescent but high adult dairy intake (OR 1.10, 95% CI 0.80, 1.52, *n* = 48 cases). A similar pattern was observed for rectal adenoma (data not shown), but sample size was limited, and confidence intervals were wide.

### Analysis stratified by age at diagnosis, family history of CRC, reason for endoscopy and behavioural factors

Higher dairy intake during adolescence was associated with lower risk of both early- (<50 y) and late-diagnosed (≥50 y) rectal and advanced adenoma (≥3.15 vs. <3.15 servings/day, early-diagnosed: rectal, OR 0.73, 95% CI 0.42, 1.28, advanced OR 0.69, 95% CI, 0.42, 1.12, late-diagnosed: rectal OR 0.75, 95% CI 0.56, 1.00, advanced OR 0.86, 0.68, 1.10, Table 3). However, the number of early-diagnosed rectal and advanced adenoma cases was limited and none of these associations did reach statistical significance. Inverse associations between dairy intake and rectal and advanced adenoma did not differ by family history of CRC (p-interaction 0.61 and 0.47, respectively).

**Table 3:** Odds ratios (OR) and 95% confidence intervals (CI) for dairy intake during high school (comparing ≥3.15 servings/day with <3.15 servings/day) and colorectal adenoma risk by age at diagnosis, family history of colorectal cancer, reason for endoscopy and behavioral factors, NHS2, 1998–2011.

	All adenoma		Rectal adenoma		Advanced adenoma	
	N Cases	OR (95% CI)*^†^	N cases	OR (95% CI)*^†^	N cases	OR (95% CI) *^†^
*Age at adenoma diagnosis*						
<50 years	267	1 (Reference)	51	1 (Reference)	80	1 (Reference)
	163	0.95 (0.75, 1.21)	25	0.73 (0.42, 1.28)	35	0.69 (0.42, 1.12)
≥50 years	1079	1 (Reference)	191	1 (Reference)	253	1 (Reference)
	730	0.99 (0.89, 1.11)	100	0.75 (0.56, 1.00)	148	0.86 (0.68, 1.10)
*Family history of CRC*						
Positive family history	299	1 (Reference)	52	1 (Reference)	69	1 (Reference)
	189	0.91 (0.73, 1.13)	28	0.79 (0.46, 1.38)	37	0.64 (0.40, 1.01)
No family history	1047	1 (Reference)	190	1 (Reference)	264	1 (Reference)
	704	0.99 (0.88, 1.11)	97	0.71 (0.53, 0.94)	146	0.86 (0.67, 1.09)
p-interaction		0.78		0.61		0.47
*Reason for endoscopy*						
Screening	934	1 (Reference)	166	1 (Reference)	213	1 (Reference)
	631	0.97 (0.86, 1.09)	77	0.68 (0.50,0.94)	115	0.75 (0.58,0.98)
Symptoms	334	1 (Reference)	63	1 (Reference)	96	1 (Reference)
	207	0.91 (0.74, 1.12)	34	0.70 (0.43, 1.15)	61	1.03 (0.70, 1.53)
p-interaction		0.32		0.92		0.82
*Smoking before age 20*						
*Body mass index (BMI)*						
BMI at age 18 <18.5	191	1 (Reference)	38	1 (Reference)	55	1 (Reference)
	133	0.98 (0.75, 1.28)	19	0.60 (0.30, 1.17)	28	0.90 (0.51, 1.58)
BMI at age 18 from 18.5 to <21	568	1 (Reference)	114	1 (Reference)	134	1 (Reference)
	378	0.94 (0.80, 1.10)	58	0.81 (0.56, 1.18)	76	0.71 (0.52, 0.97)
BMI at age 18 from 21 to <23	300	1 (Reference)	44	1 (Reference)	71	1 (Reference)
	197	1.00 (0.81, 1.24)	27	0.71 (0.41, 1.25)	48	1.16 (0.73, 1.84)
BMI at age 18 ≥23	287	1 (Reference)	46	1 (Reference)	73	1 (Reference)
	185	1.02 (0.82, 1.27)	21	0.67 (0.37, 1.20)	31	0.66 (0.40, 1.09)
p-interaction		0.83		0.49		0.12
*Physical activity 9th–12th grades*						
<median 42.5 MET-hours/week	706	1 (Reference)	132	1 (Reference)	173	1 (Reference)
	416	0.94 (0.82, 1.09)	49	0.53 (0.37, 0.78)	91	0.84 (0.62, 1.12)
≥median 42.5 MET-hours/week	602	1 (Reference)	105	1 (Reference)	148	1 (Reference)
	456	1.00 (0.86, 1.15)	73	0.98 (0.69, 1.40)	88	0.77 (0.56, 1.05)
p-interaction		0.37		0.25		0.88
Did not smoke before age 20	996	1 (Reference)	177	1 (Reference)	237	1 (Reference)
	675	0.96 (0.85, 1.07)	92	0.71 (0.53, 0.95)	139	0.85 (0.67, 1.09)
Smoked before age 20	350	1 (Reference)	65	1 (Reference)	96	1 (Reference)
	218	1.01 (0.82, 1.24)	33	0.79 (0.47, 1.32)	44	0.69 (0.44, 1.07)
p-interaction		0.87		0.66		0.79

*Comparing ≥3.15 servings/day with <3.15 servings/day.

^†^Adjusted for age, time period of endoscopy, number of reported endoscopies, time in years since most recent endoscopy, reason for current endoscopy, BMI at age 18 years (kg/m^2^, continuous), current physical activity (MET-hours/week, continuous), physical activity during 9th–12th grades (MET-hours/week, continuous), nondrinker, alcohol intake (g/d, continuous), pack-years of smoking (continuous), regular aspirin use (yes/no), menopausal status (premenopausal, postmenopausal no hormone use, postmenopausal current hormone use), total calories during high school (kcal/day, continuous), unprocessed red meat and processed meat during high school (g/day, continuous), adult calorie (kcal, continuous) and total dairy intake (g/day, continuous), family history denotes family history in first degree relatives.

There was no indication for meaningful interactions in the association between adolescent dairy intake with total, rectal or advanced adenoma by reason for endoscopy (screening vs. symptoms, all p-interaction > 0.32), BMI at age 18 years (all p-interaction > 0.12), physical activity during adolescence (all p-interaction > 0.25), or smoking before age 20 years (all p-interaction > 0.66).

## Discussion

In this large cohort of younger women, higher dairy intake during adolescence was associated with lower risk of rectal and advanced adenoma, but not proximal, distal or non-advanced adenoma. Inverse associations for rectal and advanced adenoma were independent of adult intake and did not differ considerably by age at adenoma diagnosis (early-diagnosed <50 y vs. late-diagnosed ≥50 y).

Cohort studies have consistently found an inverse association between higher intake of dairy or calcium during adulthood and risk of CRC, and the 2017 report of the World Cancer Research Fund (WCRF)/American Institute for Cancer Research (AICR) concluded that there is “strong evidence” that “consuming dairy products or taking calcium supplements decreases CRC risk”.^26^ A dose–response meta-analysis concluded that calcium intake during adulthood was inversely associated with risk of colorectal adenoma (RR per 300 mg/day 0.95, 95% CI 0.92, 0.98), with stronger associations for high-risk adenoma [≥1 cm in diameter, (tubulo)villous histology, dysplasia, or multiplicity].^28^ Prospective studies on the relationship between adult dairy intake and colorectal adenoma have also observed moderate inverse associations, but in several studies associations did not reach statistical significance.^31–33^

In contrast, little is known about the role of dairy or calcium intake during adolescence on colorectal neoplasia. Extrapolating CRC risk factors in mid-to-late adulthood to adolescence is likely inappropriate because nutritional requirements and physiology differ during different stages in life, possibly altering exposure-cancer associations.^34^ For example, puberty, a period of accelerated growth, is characterised by a physiological, and obesity unrelated, decrease in insulin sensitivity and increase in growth factor levels, such as insulin-like growth factor 1 (IGF1), possibly increasing susceptibility to environmental factors with an adverse effect on insulin metabolism.^35,36^ To our knowledge, this is the first comprehensive analysis on the association between adolescent dairy intake and colorectal adenoma. We are only aware of two previous studies relating early-life dairy intake to later risk of CRC. In the British Boyd Orr cohort, an estimate for dairy consumption during childhood was available from household food inventories for 4999 children born in the 1920s or 1930s who had been followed for up to 65 years. In that study, based on an analysis including 76 CRC cases, a positive association between childhood dairy consumption and later risk of CRC was observed.^11^ The NIH-AARP Diet and Health Study, a large prospective cohort of 292, 737 men and women who were between 50 and 71 years old at recruitment in 1995 and 1996, assessed dietary intake at ages 12-13 via a 37-item food frequency questionnaire retrospectively. Higher milk intake during adolescence was inversely associated with risk of colon cancer, but associations were substantially attenuated after taking adult dairy intake into account.^10^ However, these studies did not assess potential confounding with other relevant early-life behavioural factors or used a comprehensive and validated dietary instrument to assess exposure.

Several biological plausible mechanisms support a role of dairy intake during adolescence and colorectal neoplasia. First, dairy products are a rich source for dietary calcium, thus observed inverse associations may at least in part be mechanistically, i.e., locally, explained by the ability of calcium to bind to secondary bile-acids and ionised fatty acids, which can damage colon cells, via formation of insoluble calcium soaps.^37^ Second, calcium plays an important role in multiple signalling pathways and as such may alter cell differentiation and apoptosis.^38^ It has been suggested that calcium may act favourably on the APC/β-catenin signalling pathway by decreasing expression of β-catenin and increasing expression of APC and E-cadherin. Of relevance to our study, modifications to this pathway can be induced by high insulin and IGF1, and often occur during early stages of CRC development.^39^ Third, the calcium-sensing receptor (CaSR) is down-regulated in CRC, indicating that CaSR may act as a potential tumour suppressor in the colorectum,^40^ providing additional support for a potential causal link between calcium and CRC development. Finally, recent data also suggest that yogurt intake may have a beneficial effect on the composition of the gut microbiota.^41^ In animal models, yogurt intake has impeded promotion and progression of CRC.^42^ However, only few prospective cohort studies investigated the association between consumption of yogurt or fermented dairy products and CRC incidence, and results were inconclusive.^31,32,43–47^ In the European Prospective Investigation into Cancer and Nutrition (EPIC) an inverse association between yogurt intake and risk of CRC was observed in categorical (i.e. highest vs. lowest quartile), but not in linear models.^48^

In our study, inverse associations between adolescent dairy and risk of rectal and advanced adenoma were attenuated after adjustment for dairy calcium. Furthermore, associations between dairy and non-dairy calcium intake during adolescence and adenoma were similar, albeit weaker, to those observed for dairy intake. As dairy consumption was the major source for dietary calcium during 1960–80 when the participants were attending high school, it is difficult to assess associations for calcium vs. dairy products (particularly from milk) separately. Furthermore, dairy is also a good source of other potentially beneficial micronutrients, such as vitamin D, vitamin B12, zinc, or probiotic bacteria in fermented dairy products such as yogurt, and protein, which may also play a beneficial role during adolescence.^49^ Another possible explanation for our observed associations may be better recall of dairy intake, particularly milk (a habitual food) than other non-dairy calcium sources.^17^ In our joint analysis, it appeared that inverse associations for advanced adenoma were stronger for adolescent dairy than adult dairy intake. However, differences in physiology, metabolism, nutritional requirements, and sources of calcium between adolescents (mainly dairy) and adults (e.g., during adulthood, besides dairy other sources for calcium included calcium fortified foods and 48% of NHS2 participants were also taking calcium supplements) may at least in part explain these findings. In our previous study in the NHS2, low calcium intake (≤500 mg/day) during adulthood was associated with higher risk of colorectal adenoma.^50^ Furthermore, in our analysis, associations for dairy intake during high school appeared to be independent of dairy intake during adulthood, suggesting that higher dairy intake during both adolescence and adulthood may be independently involved in colorectal carcinogenesis. Moreover, correlation between dairy intake during adolescence and adulthood was 0.38, further reducing the possibility that dairy intake during adulthood may explain our findings.

When stratified by age at diagnosis (<50 vs. ≥50), inverse associations were observed for both early- and late-diagnosed rectal and advanced adenoma, suggesting, for the first time, that early-life dietary factors, such as dairy, the major dietary source for calcium in adolescents, may be involved in the development of both early- and late-diagnosed rectal and advanced adenoma. Interestingly, in the U.S., the recent increase in early-onset CRC incidence is primarily driven by a disproportional increase in rectal cancers.^3^ Moreover, there is evidence suggesting that associations between adult calcium or dairy intake and CRC may be stronger for distally located cancers. For example, the “Pooling Project of Prospective Studies of Diet and Cancer”, which included 10 prospective studies, found (with little evidence for heterogeneity) that higher milk intake was significantly associated with lower risk of distal colon (*p*-trend < 0.001) and rectal (*p*-trend 0.02), but not proximal colon cancers.^5^ In addition, it is possible that for advanced adenoma located in the rectum inverse associations may be even stronger than those observed for rectal and advanced adenoma separately. However, due to limited sample size, we were not able to examine rectal advanced adenoma as a separate outcome.

It has been suggested that the rising rates of obesity and physical inactivity in children and adolescents may contribute to the recent increases in early-onset CRC.^3^ Public health attempts to reverse these trends are ongoing but are unlikely to completely solve this problem in the near future. Dairy products, particularly milk is a major source of dietary calcium, and the 2015–2020 Dietary Guidelines for Americans recommend that children and adolescents consume 2–3 cup-equivalents of dairy per day^32^. However, at least in part due to replacement with sugary drinks, dairy intake in US adolescents and children has decreased in the past decades,^51^ and a considerable proportion of U.S. adolescents may not meet their RDA for calcium.^7–9^ Therefore, if confirmed by future studies, in addition to improving bone health, reversing concurrent trends such as the decrease in calcium intake in adolescents, may offer one approach to complement cancer prevention strategies targeting early-onset CRC.

Our study has some limitations that deserve further attention. First, the study population consisted predominantly of white female nurses, so we could not investigate associations by race, ethnicity, or in men, thus our findings may not be generalisable to the general population. However, in our studies on adult exposures, associations between dairy or calcium intake during adulthood and colorectal neoplasia observed in the NHS cohorts^50,52^ are mostly consistent with those from other more diverse populations.^5,53^ Second, adolescent dairy intake was recalled in 1998, years or decades after high school. However, as information of high school diet was collected prior to endoscopic procedures, the potential of recall bias, i.e. differential recall of participants with negative vs. positive endoscopy should be minimised. Moreover, reproducibility and validity of the HS FFQ was remarkably high for calcium and dairy intake with correlations of 0.73 and 0.84.^15,16^ Leveraging adolescent and adult exposure data from an established cohort with large sample size and long follow-up allowed us to adjust for a variety of potential confounders during both early-life and adulthood, conduct detailed analyses by subtypes of colorectal adenoma, and investigate whether adolescent intake was independent of adult dairy intake. Nevertheless, we can never exclude that some of our findings are explained by chance, particularly due to the multiple comparisons performed, although we carefully checked our results and investigated the robustness of findings in sensitivity analyses. Moreover, all exposures and statistical comparisons for this analysis were hypothesis-driven and chosen a priori, and our interpretation of findings takes biological plausibility, coherence, and consistency into account.

In conclusion, to our knowledge, this is the first study to suggest that dairy intake during adolescence is associated with lower risk of rectal and advanced adenoma, the latter representing subtypes that are more likely to progress to CRC. Although these observations warrant confirmation by other studies, our findings indicate that dairy intake during adolescence, the main source of dietary calcium, may play a role in the development of colorectal neoplasia, independent of adult intake.

## Supplementary information

Supplemental Tables 1-3

## Data Availability

Further information, including the procedures to obtain and access data from the Nurses’ Health Studies is described at https://www.nurseshealthstudy.org/researchers (contact email: nhsaccess@channing.harvard.edu).
