# Supplementary material for: Dairy intake during adolescence and risk of colorectal adenoma later in life
Source: Br J Cancer. 2021 Jan 4;124(6):1160–8. doi: 10.1038/s41416-020-01203-x (PMC7960961; doi:10.1038/s41416-020-01203-x)
Supplement: Supplementary file 1 — Supplemental Tables 1-3 [file 41416_2020_1203_MOESM1_ESM.pdf]

Supplemental table 1a. Odds Ratios (ORs) and 95% confidence intervals (95% CIs) for risk of colorectal adenoma according to milk intake during high school, NHS2 1998-2011

	Milk intake during high school				P_{trend}
	**≤ 2-4 servings/week**	**5 servings/week up to 1 serving/day**	**2-3 servings/day**	**≥4-5 servings/day**	
**All adenomas**					
N Cases	415	498	679	645	
Multivariable† plus adult diet	1 (Reference)	0.92 (0.80, 1.06)	0.99 (0.86, 1.14)	0.92 (0.80, 1.07)	0.58
**Proximal adenomas**					
N Cases	214	250	345	317	
Multivariable† plus adult diet	1 (Reference)	0.87 (0.72, 1.06)	0.95 (0.78, 1.14)	0.86 (0.70, 1.06)	0.34
**Distal adenomas**					
N Cases	167	223	283	285	
Multivariable† plus adult diet	1 (Reference)	1.08 (0.87, 1.33)	1.12 (0.90, 1.38)	1.12 (0.90, 1.40)	0.34
**Rectal adenomas**					
N Cases	71	76	127	93	
Multivariable† plus adult diet	1 (Reference)	0.80 (0.58, 1.11)	1.03 (0.75, 1.41)	0.71 (0.50, 1.02)	0.25
**Small, tubular adenomas (non-advanced)**					
N Cases	237	273	382	386	
Multivariable† plus adult diet	1 (Reference)	0.90 (0.74, 1.08)	1.01 (0.84, 1.21)	1.01 (0.83, 1.21)	0.50
**Large or villous adenomas (advanced)**					
N Cases	100	118	169	128	
Multivariable† plus adult diet	1 (Reference)	0.90 (0.68, 1.18)	1.00 (0.76, 1.32)	0.73 (0.53, 1.00)	0.12
**Single adenoma**					
N Cases	336	382	520	524	
Multivariable† plus adult diet	1 (Reference)	0.86 (0.73, 1.01)	0.92 (0.78, 1.07)	0.90 (0.76, 1.06)	0.51
**Two or more adenomas**					
N Cases	73	113	151	116	
Multivariable† plus adult diet	1 (Reference)	1.25 (0.93, 1.70)	1.35 (1.00, 1.82)	1.06 (0.76, 1.48)	0.82

†Adjusted for age, time period of endoscopy, number of reported endoscopies, time in years since most recent endoscopy and reason for current endoscopy, BMI at age 18 years (<18, 18-20.9, 21-22.9, 23-24.9, ≥25 kg/m2), current physical activity (<21, 21-<30, 30-<39, 39-<54, ≥54 MET hours/week), physical activity during 9th-12th grades (quintiles), current alcohol intake (<5, 5-9.9, 10-14.9, 15-29.9, 30+ g/d), pack-years of smoking (never, 1-4.9, 5-19.9, 20-39.9, 40+ pack-years), regular aspirin use (≥2 tablets/week vs. <2/week), menopausal status/postmenopausal hormone use (premenopausal, postmenopausal with current hormone use, postmenopausal without current hormone use), family history of colorectal cancer , total calories during high school (quintiles) and intake of unprocessed red meat and processed meat during high school (quintiles)

Plus adult diet=adult calorie (quintiles) and total milk intake (quintiles)

Supplemental table 1b. Odds Ratios (ORs) and 95% confidence intervals (95% CIs) for risk of colorectal adenoma according to yogurt intake during high school, NHS2 1998-2011

	Yogurt intake during high school			
	**never or <1 serving/month**	**1 serving/month up to 1 serving/week**	**≥1 serving/week**	**P_{trend}**
**All adenomas**				
N Cases	1753	334	125	
Multivariable† plus adult diet	1 (Reference)	0.93 (0.82, 1.06)	0.89 (0.74, 1.09)	0.29
**Proximal adenomas**				
N Cases	880	168	68	
Multivariable† plus adult diet	1 (Reference)	0.94 (0.79, 1.13)	0.97 (0.75, 1.26)	0.51
**Distal adenomas**				
N Cases	753	145	47	
Multivariable† plus adult diet	1 (Reference)	0.95 (0.79, 1.16)	0.78 (0.57, 1.06)	0.64
**Rectal adenomas**				
N Cases	294	52	18	
Multivariable† plus adult diet	1 (Reference)	0.88 (0.65, 1.21)	0.78 (0.48, 1.27)	0.46
**Small, tubular adenomas (non-advanced)**				
N Cases	981	212	73	
Multivariable† plus adult diet	1 (Reference)	1.06 (0.90, 1.25)	0.94 (0.73, 1.21)	0.50
**Large or villous adenomas (advanced)**				
N Cases	412	69	27	
Multivariable† plus adult diet	1 (Reference)	0.82 (0.63, 1.07)	0.81 (0.54, 1.21)	0.14
**Single adenoma**				
N Cases	1365	270	108	
Multivariable† plus adult diet	1 (Reference)	0.97 (0.84, 1.12)	0.97 (0.79, 1.20)	0.68
**Two or more adenomas**				
N Cases	369	62	17	
Multivariable† plus adult diet	1 (Reference)	0.85 (0.64, 1.13)	0.61 (0.37, 1.00)	0.25

†Adjusted for age, time period of endoscopy, number of reported endoscopies, time in years since most recent endoscopy and reason for current endoscopy, BMI at age 18 years (<18, 18-20.9, 21-22.9, 23-24.9, ≥25 kg/m^2), current physical activity (<21, 21-<30, 30-<39, 39-<54, ≥54 MET hours/week), physical activity during 9th-12th grades (quintiles), current alcohol intake (<5, 5-9.9, 10-14.9, 15-29.9, 30+ g/d), pack-years of smoking (never, 1-4.9, 5-19.9, 20-39.9, 40+ pack-years), regular aspirin use (≥2 tablets/week vs. <2/week), menopausal status/postmenopausal hormone use (premenopausal, postmenopausal with current hormone use, postmenopausal without current hormone use), family history of colorectal cancer , total calories during high school (quintiles) and intake of unprocessed red meat and processed meat during high school (quintiles)

Plus adult diet=adult calorie (quintiles) and yogurt intake (quintiles)

Supplemental table 1c. Odds Ratios (ORs) and 95% confidence intervals (95% CIs) for risk of colorectal adenoma according to cheese intake during high school, NHS2 1998-2011

	**Cheese intake during high school**			P_{trend}
	**≤1 serving/week**	**2-4 servings/week**	**≥5 servings/ week**	
**All adenomas**				
N Cases	327	540	1368	
Multivariable† plus adult diet	1 (Reference)	0.89 (0.77, 1.03)	0.91 (0.79, 1.03)	0.41
**Proximal adenomas**				
N Cases	157	268	700	
Multivariable† plus adult diet	1 (Reference)	0.93 (0.76, 1.14)	0.99 (0.82, 1.19)	0.70
**Distal adenomas**				
N Cases	146	227	584	
Multivariable† plus adult diet	1 (Reference)	0.83 (0.67, 1.03)	0.84 (0.69, 1.03)	0.29
**Rectal adenomas**				
N Cases	56	96	215	
Multivariable† plus adult diet	1 (Reference)	0.95 (0.68, 1.33)	0.85 (0.62, 1.17)	0.26
**Small, tubular adenomas (non-advanced)**				
N Cases	183	320	774	
Multivariable† plus adult diet	1 (Reference)	0.93 (0.77, 1.13)	0.88 (0.74, 1.06)	0.19
**Large or villous adenomas (advanced)**				
N Cases	79	120	315	
Multivariable† plus adult diet	1 (Reference)	0.83 (0.62, 1.11)	0.89 (0.68, 1.16)	0.84
**Single adenoma**				
N Cases	257	426	1077	
Multivariable† plus adult diet	1 (Reference)	0.90 (0.77, 1.06)	0.91 (0.78, 1.06)	0.44
**Two or more adenomas**				
N Cases	67	109	277	
Multivariable† plus adult diet	1 (Reference)	0.87 (0.63, 1.18)	0.91 (0.68, 1.21)	0.85

†Adjusted for age, time period of endoscopy, number of reported endoscopies, time in years since most recent endoscopy and reason for current endoscopy, BMI at age 18 years (<18, 18-20.9, 21-22.9, 23-24.9, ≥25 kg/m^2), current physical activity (<21, 21-<30, 30-<39, 39-<54, ≥54 MET hours/week), physical activity during 9th-12th grades (quintiles), current alcohol intake (<5, 5-9.9, 10-14.9, 15-29.9, 30+ g/d), pack-years of smoking (never, 1-4.9, 5-19.9, 20-39.9, 40+ pack-years), regular aspirin use (≥2 tablets/week vs. <2/week), menopausal status/postmenopausal hormone use (premenopausal, postmenopausal with current hormone use, postmenopausal without current hormone use), family history of colorectal cancer , total calories during high school (quintiles) and intake of unprocessed red meat and processed meat during high school (quintiles)

Plus adult diet=adult calorie (quintiles) and cheese intake (quintiles)

Supplemental table 2a. Odds Ratios (ORs) and 95% confidence intervals (95% CIs) for risk of colorectal adenoma according high-fat dairy intake during high school, NHS2 1998-2011

	High-fat dairy intake during high school (servings/day)					
	**Q1**	**Q2**	**Q3**	**Q4**	**Q5**	**P_{trend}**
**All adenomas**						
N Cases	390	477	437	495	440	
Multivariable† plus adult diet	1 (Reference)	1.07 (0.93, 1.24)	0.97 (0.84, 1.12)	1.03 (0.89, 1.19)	0.96 (0.82, 1.12)	0.43
**Proximal adenomas**						
N Cases	194	238	216	261	219	
Multivariable† plus adult diet	1 (Reference)	1.07 (0.88, 1.31)	0.95 (0.78, 1.17)	1.08 (0.88, 1.32)	0.96 (0.77, 1.19)	0.75
**Distal adenomas**						
N Cases	163	211	185	206	194	
Multivariable† plus adult diet	1 (Reference)	1.15 (0.93, 1.43)	1.00 (0.80, 1.25)	1.05 (0.84, 1.32)	1.05 (0.83, 1.33)	0.97
**Rectal adenomas**						
N Cases	73	61	81	92	60	
Multivariable† plus adult diet	1 (Reference)	0.73 (0.51, 1.03)	0.95 (0.69, 1.32)	1.01 (0.72, 1.40)	0.66 (0.45, 0.96)	0.23
**Small, tubular adenomas (non-advanced)**						
N Cases	224	269	243	276	266	
Multivariable† plus adult diet	1 (Reference)	1.04 (0.86, 1.25)	0.92 (0.76, 1.12)	0.99 (0.82, 1.20)	1.01 (0.82, 1.24)	0.95
**Large or villous adenomas (advanced)**						
N Cases	105	97	98	122	94	
Multivariable† plus adult diet	1 (Reference)	0.81 (0.61, 1.08)	0.81 (0.61, 1.09)	0.95 (0.71, 1.27)	0.76 (0.55, 1.05)	0.41
**Single adenoma**						
N Cases	306	382	338	387	350	
Multivariable† plus adult diet	1 (Reference)	1.09 (0.93, 1.28)	0.95 (0.81, 1.13)	1.02 (0.86, 1.20)	0.95 (0.80, 1.14)	0.38
**Two or more adenomas**						
N Cases	80	89	96	104	85	
Multivariable† plus adult diet	1 (Reference)	1.00 (0.73, 1.36)	1.06 (0.78, 1.44)	1.09 (0.80, 1.48)	0.98 (0.70, 1.37)	0.96

†Adjusted for age, time period of endoscopy, number of reported endoscopies, time in years since most recent endoscopy and reason for current endoscopy, BMI at age 18 years (<18, 18-20.9, 21-22.9, 23-24.9, ≥25 kg/m^2), current physical activity (<21, 21-<30, 30-<39, 39-<54, ≥54 MET hours/week), physical activity during 9th-12th grades (quintiles), current alcohol intake (<5, 5-9.9, 10-14.9, 15-29.9, 30+ g/d), pack-years of smoking (never, 1-4.9, 5-19.9, 20-39.9, 40+ pack-years), regular aspirin use (≥2 tablets/week vs. <2/week), menopausal status/postmenopausal hormone use (premenopausal, postmenopausal with current hormone use, postmenopausal without current hormone use), family history of colorectal cancer , total calories during high school (quintiles) and intake of unprocessed red meat and processed meat during high school (quintiles)

Plus adult diet=adult calorie (quintiles) and high-fat dairy intake (quintiles)

Supplemental table 2b. Odds Ratios (ORs) and 95% confidence intervals (95% CIs) for risk of colorectal adenoma according low-fat dairy intake during high school, NHS2 1998-2011

	Low-fat dairy intake during high school (servings/day)					
	**Q1**	**Q2**	**Q3**	**Q4**	**Q5**	**P_{trend}**
**All adenomas**						
N Cases	314	728	384	386	427	
Multivariable† plus adult diet	1 (Reference)	1.00 (0.87, 1.15)	0.93 (0.80, 1.09)	0.87 (0.75, 1.02)	1.02 (0.87, 1.20)	0.57
**Proximal adenomas**						
N Cases	168	368	186	194	212	
Multivariable† plus adult diet	1 (Reference)	0.94 (0.78, 1.14)	0.84 (0.68, 1.05)	0.83 (0.67, 1.03)	0.96 (0.77, 1.19)	0.80
**Distal adenomas**						
N Cases	134	309	170	169	177	
Multivariable† plus adult diet	1 (Reference)	1.01 (0.82, 1.25)	0.99 (0.78, 1.25)	0.91 (0.72, 1.15)	1.02 (0.80, 1.30)	0.91
**Rectal adenomas**						
N Cases	55	123	63	57	69	
Multivariable† plus adult diet	1 (Reference)	0.98 (0.71, 1.36)	0.89 (0.62, 1.29)	0.74 (0.51, 1.09)	0.97 (0.66, 1.41)	0.98
**Small, tubular adenomas (non-advanced)**						
N Cases	162	419	216	226	255	
Multivariable† plus adult diet	1 (Reference)	1.11 (0.92, 1.34)	1.01 (0.81, 1.25)	0.98 (0.79, 1.21)	1.17 (0.94, 1.45)	0.24
**Large or villous adenomas (advanced)**						
N Cases	88	167	83	88	90	
Multivariable† plus adult diet	1 (Reference)	0.85 (0.65, 1.10)	0.75 (0.55, 1.02)	0.74 (0.54, 1.01)	0.80 (0.58, 1.11)	0.58
**Single adenoma**						
N Cases	237	573	298	312	343	
Multivariable† plus adult diet	1 (Reference)	1.04 (0.89, 1.22)	0.95 (0.79, 1.14)	0.92 (0.77, 1.10)	1.06 (0.89, 1.28)	0.45
**Two or more adenomas**						
N Cases	75	147	82	72	78	
Multivariable† plus adult diet	1 (Reference)	0.88 (0.66, 1.17)	0.88 (0.63, 1.22)	0.74 (0.53, 1.04)	0.87 (0.61, 1.23)	0.72

†Adjusted for age, time period of endoscopy, number of reported endoscopies, time in years since most recent endoscopy and reason for current endoscopy, BMI at age 18 years (<18, 18-20.9, 21-22.9, 23-24.9, ≥25 kg/m^2), current physical activity (<21, 21-<30, 30-<39, 39-<54, ≥54 MET hours/week), physical activity during 9th-12th grades (quintiles), current alcohol intake (<5, 5-9.9, 10-14.9, 15-29.9, 30+ g/d), pack-years of smoking (never, 1-4.9, 5-19.9, 20-39.9, 40+ pack-years), regular aspirin use (≥2 tablets/week vs. <2/week), menopausal status/postmenopausal hormone use (premenopausal, postmenopausal with current hormone use, postmenopausal without current hormone use), family history of colorectal cancer , total calories during high school (quintiles) and intake of unprocessed red meat and processed meat during high school (quintiles)

Plus adult diet=adult calorie (quintiles) and low-fat dairy intake (quintiles)

Supplemental table 3a. Odds Ratios (ORs) and 95% confidence intervals (95% CIs) for risk of colorectal adenoma according to dairy intake during high school adjusted for total calcium during high school, NHSII 1998-2011

	Dairy intake during high school (servings/day)					P_{trend}
	**Q1**	**Q2**	**Q3**	**Q4**	**Q5**	
**All adenomas**						
N Cases	446	420	480	476	417	
Multivariable plus total calcium and adult diet†	1 (Reference)	0.98 (0.83, 1.15)	1.07 (0.88, 1.31)	0.99 (0.78, 1.26)	0.99 (0.75, 1.30)	0.79
**Proximal adenomas**						
N Cases	217	215	226	264/	206	
Multivariable plus total calcium and adult diet†	1 (Reference)	1.05 (0.84, 1.31)	1.08 (0.82, 1.44)	1.19 (0.85, 1.67)	1.12 (0.75, 1.66)	0.63
**Distal adenomas**						
N Cases	195	185	200	195	184	
Multivariable plus total calcium and adult diet†	1 (Reference)	0.99 (0.78, 1.26)	1.05 (0.79, 1.42)	0.99 (0.69, 1.42)	1.04 (0.69, 1.58)	0.85
**Rectal adenomas**						
N Cases	76	63	103	72	53	
Multivariable plus total calcium and adult diet†	1 (Reference)	0.86 (0.60, 1.23)	1.28 (0.84, 1.96)	0.78 (0.45, 1.35)	0.59 (0.31, 1.11)	0.03
**Small, tubular adenomas (non-advanced)**						
N Cases	239	243	263	285	248	
Multivariable plus total calcium and adult diet†	1 (Reference)	1.09 (0.88, 1.35)	1.15 (0.89, 1.50)	1.15 (0.84, 1.59)	1.16 (0.80, 1.68)	0.58
**Large or villous adenomas (advanced)**						
N Cases	114	97	122	99	84	
Multivariable plus total calcium and adult diet†	1 (Reference)	0.89 (0.65, 1.21)	1.10 (0.75, 1.60)	0.84 (0.51, 1.37)	0.81 (0.46, 1.43)	0.39

†Adjusted for age, time period of endoscopy, number of reported endoscopies, time in years since most recent endoscopy and reason for current endoscopy, BMI at age 18 years (<18, 18-20.9, 21-22.9, 23-24.9, ≥25 kg/m^2), current physical activity (<21, 21-<30, 30-<39, 39-<54, ≥54 MET hours/week), physical activity during 9th-12th grades (quintiles), current alcohol intake (<5, 5-9.9, 10-14.9, 15-29.9, 30+ g/d), pack-years of smoking (never, 1-4.9, 5-19.9, 20-39.9, 40+ pack-years), regular aspirin use (≥2 tablets/week vs. <2/week), menopausal status/postmenopausal hormone use (premenopausal, postmenopausal with current hormone use, postmenopausal without current hormone use), family history of colorectal cancer , total calories during high school (quintiles) and intake of unprocessed red meat and processed meat during high school (quintiles), total calcium intake during high school (quintiles), adult calorie (quintiles) and total dairy intake (quintiles)

Supplemental table 3b. Odds Ratios (ORs) and 95% confidence intervals (95% CIs) for risk of colorectal adenoma according to dairy intake during high school adjusted for dairy calcium during high school, NHSII 1998-2011

	Dairy intake during high school (servings/day)					P_{trend}
	**Q1**	**Q2**	**Q3**	**Q4**	**Q5**	
**All adenomas**						
N Cases	446	420	480	476	417	
Multivariable plus dairy calcium and adult diet†	1 (Reference)	0.99 (0.84, 1.17)	1.08 (0.88, 1.33)	1.00 (0.78, 1.28)	1.00 (0.75, 1.33)	0.81
**Proximal adenomas**						
N Cases	217	215	226	264	206	
Multivariable plus dairy calcium and adult diet†	1 (Reference)	1.03 (0.81, 1.30)	1.04 (0.78, 1.39)	1.12 (0.79, 1.60)	1.05 (0.70, 1.57)	0.87
**Distal adenomas**						
N Cases	195	185	200	195	184	
Multivariable plus dairy calcium and adult diet†	1 (Reference)	1.01 (0.78, 1.30)	1.08 (0.79, 1.47)	1.02 (0.70, 1.49)	1.09 (0.71, 1.69)	0.69
**Rectal adenomas**						
N Cases	76	63	103	72	53	
Multivariable plus dairy calcium and adult diet†	1 (Reference)	0.91 (0.63, 1.33)	1.36 (0.87, 2.13)	0.83 (0.47, 1.46)	0.64 (0.33, 1.22)	0.04
**Small, tubular adenomas (non-advanced)**						
N Cases	239	243	263	285	248	
Multivariable plus dairy calcium and adult diet†	1 (Reference)	1.07 (0.86, 1.34)	1.11 (0.84, 1.45)	1.09 (0.78, 1.52)	1.10 (0.75, 1.61)	0.79
**Large or villous adenomas (advanced)**						
N Cases	114	97	122	99	84	
Multivariable plus dairy calcium and adult diet†	1 (Reference)	0.92 (0.67, 1.28)	1.18 (0.78, 1.76)	0.93 (0.55, 1.55)	0.88 (0.49, 1.59)	0.53

†Adjusted for age, time period of endoscopy, number of reported endoscopies, time in years since most recent endoscopy and reason for current endoscopy, BMI at age 18 years (<18, 18-20.9, 21-22.9, 23-24.9, ≥25 kg/m^2), current physical activity (<21, 21-<30, 30-<39, 39-<54, ≥54 MET hours/week), physical activity during 9th-12th grades (quintiles), current alcohol intake (<5, 5-9.9, 10-14.9, 15-29.9, 30+ g/d), pack-years of smoking (never, 1-4.9, 5-19.9, 20-39.9, 40+ pack-years), regular aspirin use (≥2 tablets/week vs. <2/week), menopausal status/postmenopausal hormone use (premenopausal, postmenopausal with current hormone use, postmenopausal without current hormone use), family history of colorectal cancer , total calories during high school (quintiles) and intake of unprocessed red meat and processed meat during high school (quintiles), dairy calcium intake during high school (quintiles), adult calorie (quintiles) and total dairy intake (quintiles)

Supplemental table 3c. Odds Ratios (ORs) and 95% confidence intervals (95% CIs) for risk of colorectal adenoma according to dairy intake during high school adjusted for non-dairy calcium during high school, NHSII 1998-2011

	Dairy intake during high school (servings/day)					P_{trend}
	**Q1**	**Q2**	**Q3**	**Q4**	**Q5**	
**All adenomas**						
N Cases	446	420	480	476	417	
Multivariable plus non-dairy calcium and adult diet†	1 (Reference)	0.89 (0.77, 1.03)	0.96 (0.83, 1.12)	0.89 (0.76, 1.04)	0.89 (0.75, 1.06)	0.27
**Proximal adenomas**						
N Cases	217	215	226	264	206	
Multivariable plus non-dairy calcium and adult diet†	1 (Reference)	0.95 (0.78, 1.16)	0.96 (0.77, 1.18)	1.04 (0.84, 1.29)	0.97 (0.76, 1.24)	0.85
**Distal adenomas**						
N Cases	195	185	200	195	184	
Multivariable plus non-dairy calcium and adult diet†	1 (Reference)	0.90 (0.73, 1.12)	0.92 (0.74, 1.16)	0.85 (0.67, 1.07)	0.91 (0.71, 1.17)	0.44
**Rectal adenomas**						
N Cases	76	63	103	72	53	
Multivariable plus non-dairy calcium and adult diet†	1 (Reference)	0.77 (0.55, 1.08)	1.15 (0.83, 1.59)	0.72 (0.50, 1.04)	0.57 (0.38, 0.87)	0.01
**Small, tubular adenomas (non-advanced)**						
N Cases	239	243	263	285	248	
Multivariable plus non-dairy calcium and adult diet†	1 (Reference)	0.96 (0.79, 1.16)	0.99 (0.81, 1.21)	1.00 (0.81, 1.23)	1.03 (0.82, 1.29)	0.67
**Large or villous adenomas (advanced)**						
N Cases	114	97	122	99	84	
Multivariable plus non-dairy calcium and adult diet†	1 (Reference)	0.82 (0.62, 1.08)	0.97 (0.72, 1.29)	0.72 (0.52, 0.99)	0.67 (0.48, 0.95)	0.02

†Adjusted for age, time period of endoscopy, number of reported endoscopies, time in years since most recent endoscopy and reason for current endoscopy, BMI at age 18 years (<18, 18-20.9, 21-22.9, 23-24.9, ≥25 kg/m^2), current physical activity (<21, 21-<30, 30-<39, 39-<54, ≥54 MET hours/week), physical activity during 9th-12th grades (quintiles), current alcohol intake (<5, 5-9.9, 10-14.9, 15-29.9, 30+ g/d), pack-years of smoking (never, 1-4.9, 5-19.9, 20-39.9, 40+ pack-years), regular aspirin use (≥2 tablets/week vs. <2/week), menopausal status/postmenopausal hormone use (premenopausal, postmenopausal with current hormone use, postmenopausal without current hormone use), family history of colorectal cancer , total calories during high school (quintiles) and intake of unprocessed red meat and processed meat during high school (quintiles), non-dairy calcium intake during high school (quintiles), adult calorie (quintiles) and total dairy intake (quintiles)
